# Bipolar disorders in Nigeria: a mixed-methods study of patients, family caregivers, clinicians, and the community members’ perspectives

**DOI:** 10.1186/s40345-022-00276-2

**Published:** 2023-01-07

**Authors:** Dung Ezekiel Jidong, M. Ishrat Husain, Tarela J. Ike, Nusrat Husain, Maigari Yusufu Taru, Nwoga Charles Nnaemeka, Christopher Francis, David B. Jack, Shadrack B. Mwankon, Siqi Xue, Juliet Y. Pwajok, Pam P. Nyam, Maisha Murshed

**Affiliations:** 1 Department of Psychology, Nottingham Trent University, 50 Shakespeare Street, Nottingham, NG1 4FQ UK; 2 Campbell Family Mental Health Research Institute, Centre for Addiction and Mental Health, Toronto, Canada; 3 Department of Criminology & Sociology, Teesside University, Middlesbrough, UK; 4 Department of Psychiatry, University of Manchester, Manchester, UK; 5 Department of Psychiatry, Jos University Teaching Hospital, Jos, Nigeria; 6 Global Mental Health, Dung Jidong Foundation, Jos, Nigeria; 7 Department of Psychology, University of Jos, Jos, Nigeria

**Keywords:** Bipolar disorders, Caregivers, Patients, Mental health, Psychological intervention, Nigeria

## Abstract

**Background:**

Bipolar Disorders (BDs) are chronic mental health disorders that often result in functional impairment and contribute significantly to the disability-adjusted life years (DALY). BDs are historically under-researched compared to other mental health disorders, especially in Sub-Saharan Africa and Nigeria.

**Design:**

We adopted a mixed-methods design. Study 1 examined the public knowledge of BDs in relation to sociodemographic outcomes using quantitative data whilst Study 2 qualitatively assessed the lived experiences of patients with BDs, clinicians, and family caregivers.

**Methods:**

In Study 1, a non-clinical sample of *n* = 575 participants responded to a compact questionnaire that examined their knowledge of BDs and how they relate to certain sociodemographic variables. One-way ANOVA was used to analyse quantitative data. Study 2 interviewed *N* = 15 participants (*n* = 5 patients with BDs; *n* = 7 clinicians; *n* = 3 family caregivers). These semi-structured interviews were audio-recorded, transcribed, and thematically analysed.

**Results:**

In Study 1, findings showed no statistically significant differences, suggesting low awareness of BDs, especially among vulnerable populations such as young people and older adults. However, there was a trajectory in increased knowledge of BDs among participants between the ages of 25–44 years and part-time workers compared to other ages and employment statuses. In Study 2, qualitative findings showed that BDs are perceived to be genetically and psycho-socially induced by specific lived experiences of patients and their family caregivers. Although psychotropic medications and psychotherapy are available treatment options in Nigeria, cultural and religious beliefs were significant barriers to treatment uptake.

**Conclusions:**

This study provides insight into knowledge and beliefs about BDs, including the lived experiences of patients with BDs, their caregivers and clinicians in Nigeria. It highlights the need for further studies assessing Nigeria's feasibility and acceptability of culturally adapted psychosocial interventions for patients with BDs.

## Introduction

Mood disorders are psychological illnesses that affect emotions and behaviours. Prominent among mood disorders are bipolar disorders (BDs), which represent a significant disease burden (Husain et al. 2017) and account for 7% of the disability-adjusted life years (DALY) caused by mental disorders (Whiteford et al. 2013). The recurrent biphasic mood states of mania or hypomania and depression that characterise BDs cause impaired functioning and reduced quality of life. The World Mental Health Survey Initiative found that BDs affect more than 1% of the general population and have an estimated 12-month and lifetime prevalence rates of 1.5% and 2.4% respectively (Merikangas et al. 2011). A recent study reported a lifetime prevalence of BDs to be 1.72% of the general population in England (Humpston et al. 2021). According to the study, 4 out of 10 persons with a probable diagnosis of BDs received no mental health care within the preceding 12 months. Compared to the general population, individuals with BDs tend to have a significantly higher rate of associated suicide mortality (Crump et al. 2013). Within the last decade, these mortality rates have substantially increased (Lomholt et al. 2019), suggesting the need for more targeted research to address the unresolved needs of individuals living with BDs. A recent meta-analysis found that compared to the general population, patients with BDs had reduced life expectancy with about 13 years of potential life loss (Chan et al. 2022).

Given their comorbid potentiality with other mental illnesses, including substance use disorders, BDs significantly burden health care services. While the fifth edition of the Diagnostic and Statistical Manual of Mental Disorders (DSM 5) suggests that BDs less frequently occur in low-income countries than in high-income countries (APA, 2013), it is still prevalent in many parts of Africa. A systematic review of five studies investigating the prevalence of BDs among the general African population reported a range of 0.1–1.83% (Esan & Esan, 2016). In Nigeria, the prevalence of BDs has been reported as 0.1% among the general population (Gureje et al. 2008). A possible reason for the low prevalence might be due to methodological pitfalls in published studies, as the authors utilised only the World Mental Health version of the Composite International Diagnostic Interview (WMH-CIDI). It has been recommended that in low-income countries, combining the Key Informant Method and CIDI were more likely to aid the diagnosis of mental disorders (Shibre et al. 2002).

A systematic review of literature from two decades (1992–2012) on public attitudes and internalised stigma of BDs found inconsistency and considerable gaps in the literature (Ellison et al. 2013). Of note, no study emanated from Africa from all 25 included peer-reviewed studies. In a study assessing public beliefs and attitudes towards BDs disorders in a UK sample, BDs were believed to have both biomedical and psychosocial origins; however, they were associated with positive stereotypes, such as the belief that individuals with BDs are intelligent and creative (Ellison et al. 2015). Discrimination against mental illness in Africa has often been documented in published literature (Jidong et al. 2021a). Such discriminatory acts among the general population sometimes arise from misinformation about mental disorders (Jidong et al. 2021d). In Nigeria, some studies have explored lived experiences of maternal anxiety and depression (e.g. Jidong et al. 2021b); however, there is a dearth of literature investigating how the general public views individuals with BDs. A previous study of first-degree relatives of patients with schizophrenia and major affective disorders with psychotic symptoms in psychiatric hospitals in Nigeria found that contrary to relatives of sickle cell patients who mostly cited genetic components as the root cause of the patients’ illness, caregivers of patients with psychotic illnesses had more religious-influenced opinions about the aetiology of these illnesses (Oheari and Fido, 2001; Jidong et al. 2021a).

While religion-influenced opinions are observed to be widespread in Nigeria, with a minute fraction tilting towards religious extremism (Onimhawo & Ottuh, 2007), it is important to note that these psychosocial stressors could interact dynamically to precipitate, perpetuate and/or exacerbate the symptoms of BDs (Jidong et al. 2021a; Jidong et al. 2022). As such, this study adopts the social zeitgeber theory of bipolar spectrum disorders (Ehlers, Frank, & Kupfer, 1988) as a viable theoretical lens for explaining the pathology of BDs. The theory opined that high-risk individuals could potentially thwart biological rhythms due to altered social zeitgebers (Boland et al. 2015). Such alterations could result in various affective symptoms expressed in extreme poles. Within the Nigerian context, several factors could potentially disrupt an individual’s social zeitgeber; prominent in extant literature are cultural beliefs about mental illness (Jidong et al. 2022) and poverty (Abdulmalik et al. 2019; Jidong et al. 2021b).

Despite the reviewed research, there are still significant gaps in understanding public knowledge and beliefs about BDs in Sub-Saharan Africa and Nigeria. Most literature on public beliefs or knowledge about mental illness in Nigeria explored mental disorders in general (Okpalauwaekwe et al. 2017; Adewuya et al. 2008; Kabir et al. 2004), while other research has focused on depression or schizophrenia. With differential public attitudes about individual mental disorders (Crisp et al. 2000), it is important to expand beyond this current knowledge, especially given the similar global prevalence rates between BDs and schizophrenia. Additionally, it is imperative to understand public views since individuals with BDs spend most of their time functioning within a social space. There appears to be a gap in the cultural or context-specific understandings of the aetiology, diagnosis and treatment of BDs in Nigeria. The present study utilised a mixed methods design to evaluate public knowledge of BDs. The lived experiences of patients with BDs, clinicians’ experiences working with BD patients and evaluation of interventions to address the BDs in Nigeria.

The quantitative methods (Study 1) hypothesised that participants would significantly differ in their knowledge of BDs based on age, gender, employment and marital status. The qualitative methods (Study 2) aimed to assess (i) the lived experiences of individuals with BDs in Nigeria and (ii) the experiences of clinicians and family caregivers supporting individuals with BDs.

## Methods

### Design

This study uses a mixed methods design integrating quantitative and qualitative approaches to investigate significant aspects of BDs across clinical and non-clinical populations in Nigeria. The Medical Research Council (MRC) recommends mixed methods studies to examine mental health topics in preparation for sustainable interventions (Craig et al. 2008). Mixed-methods also enhances the robustness of study results (Craig et al. 2008; Jidong et al. 2022).

### Study 1: participants, instrument, and data collection

In study 1, we adopted a convenience sampling technique and administered *n* = 600 questionnaires to a non-clinical sample. Of the *n* = 600 questionnaires, only *n* = 575 were returned as valid responses. Participants were recruited in major cities across Plateau state (located within the northcentral), Ekiti and Oyo states (located in the southwestern regions) of Nigeria. These participants completed a concise questionnaire in the English language that assessed their sociodemographic variables such as gender, age, employment status and marital status, among others. The other section of the questionnaire assessed their beliefs and knowledge about BDs. Specifically, knowledge of BDs was assessed using the Oxford Bipolar Knowledge Questionnaire (OBKQ), developed by Bilderbeck et al. (2016). The OBKQ consists of 10 items with four statements that utilise a 3-point Likert scale of 0 (disagree) to 2 (agree). The responses are summed up to a total composite score, ranging from 0 to 80. A higher score is indicative of better knowledge of BDs and vice versa. The scale's reliability was further established in this present study with a Cronbach’s alpha of 0.799.

### Study 1 data analysis

The quantitative data in this study were analysed using Statistical Package for Social Sciences (SPSS) version 25. Participants’ sociodemographic data were analysed using descriptive statistics to assess frequencies and percentages. All hypotheses were analysed using a One-way Analysis of Variance (ANOVA). The study’s objectives informed the choice of statistics to investigate mean differences among multiple categories of sociodemographic variables on knowledge of BDs (which was measured on an interval scale).

### Study 2: participants, interview questions and data collection

Purposive and snowball sampling techniques were adopted to recruit *n* = 15 participants, consisting of *n* = 7 clinicians, *n* = 5 patients and *n* = 3 family caregivers. Semi-structured interviews that lasted approximately 60 min each were utilised to investigate the participants’ defined meanings and lived experiences of issues around BDs. Some sample interview questions include (a) Please can you tell me your general thoughts about bipolar disorder? (b) Does family and societal impact play a role in reducing or exacerbating experiences of bipolar disorder? (c) Can you tell me what your community members think about people with previous histories of bipolar disorder (d) Do you think cultural/religious beliefs play any role in understanding bipolar disorder and its treatment? (e) What kind of health support will you get that can benefit better mental well-being of people suffering from bipolar disorder?

Similarly, these semi-structured interviews were used to explore the family caregivers' experiences of patients living with BDs and clinicians’ experiences working with patients with BDs. Three trained clinical research assistants conducted all interviews in English. All interviews were conducted face-to-face in quiet and uninterrupted consulting rooms in clinics and hospital settings. Some sample interview questions for clinicians and family caregivers include (f) What are your experiences of working with patients who are experiencing bipolar disorders? (g) What could be unique/peculiar about the patients you treat that is different from the mainstream western perspectives?

### Study 2 data analysis

The audio-recorded interviews were transcribed verbatim and analysed using thematic analysis from critical realist and socio-constructionist theoretical lenses (Harper 2011a, b). Therefore, both the realist and constructionist features in the datasets were explored. The theoretical research lenses acknowledged that participants’ lived experiences and defined meanings surrounding BD discourses are shaped by the context-specific cultural realities in the social realm, which widely depend on their beliefs and experiences (Burr 2015). In essence, the present study assumed that dominant narratives in the dataset from the participants might not be a direct representation of their realities but are influenced by the knowledge of their realities as a product of shared history, language and social space, which are instrumental to understanding BDs within the designated context (Jidong et al. 2021a, d).

### Study 1 results

Table 1 reveals a roughly equal distribution of female (49.6%) and male participants, with a minute proportion (0.3%) identifying as non-binary. The participants' ages ranged from 18 to 65, with most of the participants falling within early to mid-adulthood. Almost half of the participants (44.7%) identified as students, while the rest reported a variety of other employment statuses. Nearly two-thirds of participants (63%) reported never being married.

**Table 1:** Participants' characteristics and demographic information

	Frequency (*n)*	Percent (%)
Gender		
Male	288	50.1
Female	285	49.6
Non-binary	2	0.3
Total	575	100
Age		
18–24 years	201	35.1
25–34 years	171	29.9
35–44 years	153	26.7
45 years and above	47	8.2
Missing	3	0.5
Total	575	100
Employment status		
Working full-time	96	17.0
Working part-time	134	23.7
Unemployed and looking for a job	51	9.0
Stay-at-home parent	14	2.5
Student	257	45.5
Other	13	2.5
Missing	10	1.7
Total	575	100.0
Marital status		
Married	161	28.5
Living with a partner	21	3.7
Widowed	18	3.2
Divorced/separated	3	0.5
Never been married	362	64.1
Missing	10	1.7
Total	575	100.0

**Table 2:** ANOVA Summary Table Showing Influence of Sociodemographic Variables on Knowledge of Bipolar Disorders (KBDs)

	KBDs			
	*M* (*SD*)	*F*	*P*	*η*^*2*^
Gender		2.782	0.063	0.010
Male	56.08 (12.23)			
Female	53.78 (14.05)			
Non-binary	45.0 (12.03)			
Age		13.921	0.0001*	0.070
18–24 years	50.66 (15.28)			
25–34 years	57.85 (10.85)			
35–44 years	57.90 (9.84)			
45 years and above	52.13 (15.93)			
Employment status		3.497	0.004*	0.030
Working full-time	56.40 (11.33)			
Working part-time	57.74 (10.72)			
Unemployed	56.71 (11.80)			
Stay-at-home parent	56.93 (11.23)			
Student	52.93 (14.53)			
Other (e.g., entrepreneurs, farmers etc.)	49.54 (13.36)			
Marital Status		1.365	0.245	0.010
Married	56.53 (10.56)			
Living with a partner	56.90 (9.15)			
Widowed	57.22 (10.76)			
Divorced/separated	52.00 (15.72)			
Never been married	53.96 (14.48)			

*KBD* knowledge of bipolar disorders, *M* mean, *SD* standard deviation

* Indicates significant p value at < 0.05

A one-way ANOVA was performed to test the hypothesis that participants’ knowledge of BDs differs significantly across their gender (males, females and non-binary); see Table 2. While there were numerical differences in knowledge of BDs between males (*M* = 56.08, *S* = 12.23, females (*M* = 53.78, *S* = 14.05) and persons who identified as non-binary (*M* = 45.0, *S* = 12.03), these differences were not statistically significant, *F* (2, 572) = 2.782, *p* = 0.063.

Age-specific differences in knowledge of BDs among participants were tested with a between-groups ANOVA. The results revealed a statistically significant influence of age on knowledge of BDs, *F* (3, 568) = 13.921, *p* = 0.001, *η*^*2*^ = 0.070. 7% of the variances observed in participants’ knowledge of BDs were accounted from violating the assumption of homogeneity as indicated by Levene’s *F* test *F* (3, 568) = 7.522, *p* = 0.001. The Games-Howell multiple comparison revealed statistically significant differences in participants between 18–24 years (*M* = 50.66, *S* = 15.275) and 25–34 years (*M* = 57.85, *S* = 10.845) (*p* < 0.05), and 18–24 years (*M* = 50.66, *S* = 15.275) and 35–44 years (*M* = 57.87, *S* = 9.841) (*p* < 0.05). Statistically significant differences were not observed in other conditions (*p* > 0.05).

A one-way ANOVA was also used to test the hypothesis that the participants’ employment status would significantly influence their knowledge of BDs. Prior to conducting the one-way ANOVA, the assumption of the equality of variances was tested and satisfied via Levene’s *F* test, *F* (5, 559) = 1.975, *p* = 0.083. The one-way ANOVA showed a statistically significant influence of employment status on participants’ knowledge of BDs, *F* (5, 559) = 3.497, *p* = 0.004, *η*^*2*^ = 0.030. The differences between the various levels of employment status were examined through a Tukey’s HSD multiple comparison. The result revealed significant mean differences between part-time workers and students on knowledge of BDs (*p* = 0.006). There was no other significant difference found in the other conditions (*p* > 0.05).

Lastly, the hypothesis that participants’ knowledge of BDs will differ significantly across their marital status was tested with a one-way ANOVA. The results showed no significant influence of marital status on participants’ knowledge of BDs, *F* (4, 560) = 1.365, *p* = 0.245.

### Study 2 findings

Four themes emerged from the qualitative analyses in Study 2. These include: (i) Perceptions of BDs as being genetically and psycho-socially induced (ii) Lived experiences of patients with BDs and their caregivers’ perspectives (iii) The role of psychotropic medications and psychoeducation in treatment and (iv) Cultural and religious beliefs as barriers to the treatment of BDs. These results are presented with extracts of data verbatim supporting each theme.

(i) Perceptions of BDs as being genetically and psycho-socially induced

The dataset associated the causes of BDs with genetic factors. However, one of the interviewed service-users provided a belief perspective about the association of BDs with witchcraft and cultism:

*“Some people don’t believe that a problem can be physical, or it can be genetic. All they believe is that they try to marry it [link bipolar disorder] to witchcraft, cultism, and the rest of them. So, they don’t believe that something [bipolar disorder] can be natural […] Like my own, I was drugged with food, somebody put marijuana in my food ignorantly, and because I have a large brain it now resulted to a problem in my brain. So, these are some of the few factors that can cause bipolar disorder”* (32-year-old male with BD).

One clinician associated the cause of BDs with genetic and socio-economic challenges:

*“The genetic transmission of the trait is very high it tends to run in families, and then it also bipolar coexists with other mood disorders and others psychotic illnesses when people have stressful life event like loss of a loved ones like conflict especially arm conflict that is common in our environment like stress at office, like economy difficulties it can bring forward bipolar illness in somebody that is already genetically predisposed“* (53-year-old female clinician).

(ii) Lived experiences of patients with BDs and caregivers’ perspectives

The dataset revealed participants’ lived experiences of their expressive behaviours and how it was perceived differently in their communities. For example, two interviewed patients commented as follows:

*“I say so because I have experience what people have done to me, through betrayal, apart from betrayal, forcing me to be stigmatised, through insult and offensive words they use on me as a mad person while I am not mad. Because each time, I normally pull my cloth and walk naked on the street. So, it was my late father that was encouraging me all the time. Telling me son, you have to be yourself. Feel free in the society, feel free in the family. Feel free everywhere you find yourself. Because my father encourages me as far as he is concerned, there is no stigmatisation. As far as he is still concern” (28-year-old male patient with BD).*

*“Hmm! discrimination, at times you will be going and they will be like look at that man, people look down on you that will make someone rejected and depressed as you pass by people point at you calling you name and gossip about you in your presence, and they look down on you too much people you are close with before tend to forsake you, those that close to you will turn their back against you, nobody to talk to you, nobody to communicate to you, nobody to share your problem with”* (26-year-old male patient with BD).

Both patients above felt betrayed, discriminated against, and stigmatised. One recounted his expressive behaviours, especially with the continuous removal of his clothes and how this was strongly encouraged by his late father. However, the Nigerian cultural or context-specific attribution to the removal of his clothes might have been attributed to ‘madness’ which might not be the implication in other cultures or countries. The other patient also felt neglected and discriminated against by his friends and close associates, leading to more feelings of loneliness and depressive moods.

The lived experience of one interviewed caregiver was narrated as follows:

*“I will often tell my daughter that I sympathise with her because I look at it as if her future is gone eh I look at her in the next ten years how is she going to look like and then I look at her tomorrow if I'm not there will her senior ones [siblings] take care of her so you see I sympathise with her I sympathise with her in fact that is just the basic thing I sympathise with her, we have been spending a lot of money and everything that we have- so if there is anything, I don't have much things to say- abeg! Ku bar ni, abeg! [you should leave me please]”* (38-year-old female caregiver of a patient with BD)*.*

This caregiver shared her burden of caring for her daughter. The latter has severe BD, which she found to be very emotionally disheartening, as depicted in the above extract in which she became emotional and could not talk any further. From a slightly different perspective, another interviewed caregiver talked about unconditional love for their family member who was diagnosed with BD:

*“We that are close family members to the person [bipolar patient] we need to show love first before others will see- we need to take good care of them [bipolar patients] so that others or outsiders will see- if we bring them close to us and show them that love that is how others too will respect them for that work we do but if we neglect them that is how everybody will be looking at them any how that is it”* (33-year-old female caregiver).

(iii) The role of psychotropic medications and psychoeducation in treating patients with BDs

Findings indicated that medications and psychotherapy were prominent treatment modalities used in services where participants were recruited for this study. For example, one interviewed patient said:

*“Yes, I am a victim of the herbs I have once stopped my drugs and concentrated on the herbs and the herbs could not help me. I did even take the herbs for a long time, and I had a relapse, so I went back to my old [antipsychotic] medications. That’s how I am able to get myself, finished my studies, working well, taking care of my family and doing other things normal the way normal human beings do’’* (22-year-old female patient with BD).

An interviewed clinician placed emphasis on psychoeducation as an essential component of psychotherapy for treating BD:

*“We have psychotherapy where we engage the bipolar patients with treatment, we psycho-educate them about the nature of the illness, we detect any warning symptoms or signs they should be aware of such as irritability, over familiarity, over-generosity and so many key symptoms that we teach them to identify”* (53-year-old female clinician)*.*

Psychoeducation forms a central component of the above extract, aimed at educating BD patients on the features of their disorders and potentially self-monitoring or directing their lived experiences. Psychoeducation may be beneficial not only to the service users but also the society on how they perceive and relate with individuals with BDs in their day-to-day interactions. For example, an interviewed service user said:

*“We should show the person [BD patient] love and the society should not look down on hem for example if somebody is taking drug we should not discriminate against the person, instead they should try to come close to the person, to know what it want by making sure his taking his drug regularly, by showing of love, care, assisting, the person maybe slow in doing something they should exercise patience” (23 year old patient with BD).*

(iv) Cultural and religious beliefs as barriers to the treatment of BDs

Cultural and religious beliefs were indicated as common challenges and barriers to the uptake of mental health interventions for patients with BDs. One of the interviewed patients said:

*“They [religious healers] will tell you your solution lies in the hands of God that you can do without drugs [psychotropic medications]. God is alive, come for prayers, stop your medication, fast for some number of days. It will be well with you. They have succeeded in deceiving many patients too. You will fast and fast! At the end of the day, you will still have a relapse”* (26-years-old male patient with BD).

This patient construed religious healers as potentially unhelpful, especially when they oppose the use of medications, leading to relapse. Similarly, a clinician said:

*“Well, I have seen quite a lot of patients who have bipolar affective disorder and the major challenges we have with them is the uptake of their medications, because we have quite a number of them that have been cycled reputedly. So, they come on admission about four times in a year. They failed to comply with their medications because when they take it, and they feel better. They believe they are well, and they stop taking their medications. And then of course then, they have a relapse and they come back to the hospital again and again”* (38-year-old female clinician).

Another interviewed clinician reiterated the view that non-adherence to treatment may be linked to religious beliefs:

*“They can decide not to go for psychotherapy sessions, until they are persuaded because they have already been told by some of their religious leaders that this is spiritual problems, these are attacks from enemies within and without so all they need is prayers and deliverance. Based on our culture here in Africa and Nigeria, they hold on to those juju [fetish] things more than any other thing. So, these are some of the factors affecting our treatment”* (28-year-old female clinician).

## Discussion

Our study explored the Nigerian public's knowledge and attitudes towards BDs while examining the lived experiences of individuals diagnosed with bipolar disorder in Nigeria. We found no statistically significant differences in knowledge of BDs based on gender. A possible reason for this may be the equal access across genders to education or knowledge in Nigeria. Within the past few decades, there has been a rise in advocacy for girls’ education and other campaigns that promote access to education. The results align with a study that explored public awareness, beliefs and attitudes towards BDs in Saudi Arabia (Alosaimi et al. 2019). Using sociodemographic variables to predict public knowledge of BDs, this study showed that gender was not a significant predictor of knowledge of BDs among the general public.

Our study found age-specific differences in knowledge of BDs among the Nigerian public. Of particular importance, the results show a specific trajectory in knowledge, with participants between the ages of 25–44 years having the highest knowledge of BDs. However, a sudden decline was observed in the knowledge curve among participants 45 years and older, with the poorest knowledge observed in the early age and older adults, vulnerable age groups for BDs. Caution should be taken when interpreting this data set, as there is a relatively small sample in the 45 and older group, which calls for further study. In addition, future studies could collect data on years of education which could bring more information than employed or unemployed. These were anticipated findings as early to mid-adulthood are important developmental ages where individuals are most exposed to knowledge and awareness of several disorders.

Further, the stiff decline in the knowledge curve as age progressively increases shows the rise in mental health awareness in recent times due to public health campaigns and other strategies aimed at increasing mental health awareness in Nigeria. Although our study found age to predict public knowledge depending on the age group, this result contrasted with Alosaimi et al.’s (2019) in Saudi Arabia, which identified age as not significant predictor of public knowledge of BDs. Such inconsistencies could be traced to the differential structures aimed at increasing mental health awareness, tenable in both countries over the past decades. In Nigeria, for example, awareness of mental illness is still growing, substantially excluding older Nigerian adults from the privilege of having access to it during their active years of socialisation.

We observed that differences in knowledge of BDs were dependent on employment status, with part-time workers showing the highest knowledge of BDs. Further post-hoc analysis localised these statistically significant differences between part-time workers and participants who identified as full-time students. Admittedly, part-time workers could both work and attend school at the same time, hence, would have better exposure to resources and other activities that could potentially place them in a more advantageous position to have better knowledge of mental illnesses such as BDs when compared to full-time students who, channel most of their time and resources on their academics. We found similar knowledge of BDs across the various categories of marital status. This contrasts with previous work that found marital status to predict knowledge of bipolar disorders, with married participants having significantly lower scores when compared to single participants (Alosaimi et al. 2019).

The qualitative ambit of our study examined the lived experiences of Nigerian patients with BDs, their family members and clinicians’ perspectives. Findings showed that the causes of BDs are perceived to be genetically and psycho-socially induced by specific lived experiences of patients and caregivers or family members’ shared burdens. Although psychotropic medications and psychotherapy are available treatment options, cultural and religious beliefs were serious barriers to the uptake of biomedicine-based or Western medicine interventions. These findings are similar to a qualitative study on Nigerian cultural beliefs in which spiritual causes of mental health conditions were found to be paramount beliefs (Jidong et al. 2021a). Similarly, it has been reported that cultural and religious beliefs play a significant role in mental health treatment in Nigeria (Jidong et al. 2022). One participant in the present study reported the inadequacy of using traditional herbs in treating BDs contrary to a previous study that reported traditional medicines to help with bereavement loss, trauma and coping with mental health disorders in South Africa (Makgahlela et al. 2021).

To the best of our knowledge, this is the first community-based cross-sectional mixed methods study that examined public knowledge of BDs in relation to specific sociodemographic variables in Nigeria. The study provides insight into how the general public conceptualises and views BDs, and identifies knowledge gaps across several demographics. One of the limitations of this work is the convenient sampling technique adopted to collect data for the study's quantitative arm, which could limit the potential to generalise the findings across Nigeria as a whole. Future studies could employ randomised sampling techniques and consider exploring more nuanced but salient demographics such as religious orientation and religious leaders’ perspectives of BDs.

The study underlines a limited awareness of BDs in Nigeria, especially among the vulnerable population that might be prone to BDs, such as young people. The implications of cultural and religious beliefs for treating and managing BDs in Nigeria are also important considerations for developing locally tailored interventions. We encourage the development and feasibility testing of a culturally adapted intervention specific to patients with BDs in Nigeria. A co-developed and adapted intervention with inputs from key stakeholders such as local people with lived experiences, family caregivers and local clinicians, could lead to a scalable and low-cost to improve outcomes for individuals with BDs in Nigeria and other African settings.

## Data Availability

The datasets generated and analysed during the current study have been utilised and reported in the paper. No further data could be made public due to the confidentiality agreement with the study participants.
